# Genome-wide identification of the CLAVATA3/EMBRYO SURROUNDING REGION (CLE) family in grape (*Vitis vinifera L*.)

**DOI:** 10.1186/s12864-019-5944-2

**Published:** 2019-07-05

**Authors:** Pengfei Wang, Yongmei Wang, Fengshan Ren

**Affiliations:** Shandong Academy of Grape; Shandong Engineering Research Center for Grape Cultivation and Deep-Processing, Jinan, 250100 People’s Republic of China

**Keywords:** *CLE* gene, Environmental stimuli, CLE motif, Codon usage bias

## Abstract

**Background:**

*CLE* genes play various biological roles in plant growth and development, as well as in responses to environmental stimuli.

**Results:**

In the present study, we identified nine *CLE* genes in the grape genome using an effective identification method. We analyzed the expression profiles of grape *CLE* genes in different tissues and under environmental different stimuli. *VvCLE3* was expressed in shoot apical meristem (SAM) enriched regions, and *VvCLE6* was expressed in shoot tissue without SAM. When grapes were infected with bois noir, *VvCLE2* was up-regulated. Under ABA treatment, *VvCLE3* was down-regulated. *VvCLE6* was up-regulated under high temperature stress. We found that *VvCLE6* and *VvCLE1* were highly expressed in root tissue. In addition, we compared the characteristics of CLEs from grape and other plant species. The CLE family in *Sphagnum fallax* underwent positive selection, while the CLE family in grape underwent purifying selection. The frequency of optimal codons and codon adaptation index of rice and grape CLE family members were positively correlated with GC content at the third site of synonymous codons, indicating that the dominant evolutionary pressure acting on rice and grape *CLE* genes was mutation pressure. We also found that closely related species had higher levels of similarity in relative synonymous codon usage in *CLE* genes. The rice *CLE* family was biased toward C and G nucleotides at third codon positions. Gene duplication and loss events were also found in grape *CLE* genes.

**Conclusion:**

These results demonstrate an effective identification method for *CLE* motifs and increase the understanding of grape *CLEs*. Future research on *CLE* genes may have applications for grape breeding and cultivation to better understand root and nodulation development.

**Electronic supplementary material:**

The online version of this article (10.1186/s12864-019-5944-2) contains supplementary material, which is available to authorized users.

## Background

Small signaling peptides (SSPs) are secreted chains of amino acids that typically have five to 50 residues, and are often encoded within a longer protein sequence of about 100 to 200 amino acids called a preproprotein. SSPs can act like hormones, and their cognate receptors can transmit local and systemic signals [1]. SSP signals relay information that coordinate cell proliferation and differentiation during plant development, and SSPs often bind to corresponding families of receptors and are encoded by gene families [2]. SSPs are known to include the CLAVATA3/EMBRYO SURROUNDING REGION (CLE) and the C-TERMINALLY ENCODED PEPTIDE (CEP) families [1]. The CLE peptide family is a well-studied peptide family in plants and has been found in many plant species and some parasitic nematodes [3–11]. Thirty-two *CLE* genes been reported from *Arabidopsis thaliana* [12], while 15 and 50 *CLE* genes have been identified in tomato [13] and *Populus trichocarpa* [3], respectively.

CLEs contain a conserved 14-amino acid (aa) consensus sequence (conserved sequence motif KRXVPXGPNPLHNR), which is called the CLE motif or CLE domain [6]. However, some reports indicate that the conserved CLE motif is 12–13 aa in length [10, 12, 14]. Several studies have shown that the conserved CLE motif is the functional region of CLE peptides, and the mature CLE peptide is derived directly from the CLE motif [12]. The mature CLE peptides contain 12–13 amino acids that are proteolytically released from the precursors by a serine protease [15, 16]. Based on previous research that identified 12 amino acids as the conserved motif, the typical CLE motif has histidine (H1) or arginine (R1) at the first amino acid position. The research that indicated that the conserved motif is 13–14 amino acids long identified that the typical CLE motif has histidine (H2) or arginine (R2) at the second amino acid position [6]. Whether the 12, 13, or 14 amino acids are conserved, most typical CLE conserved motifs have three prolines [6, 17]. The 12-aa motifs contain P4, P7, and P9, while 13–14 aa motifs often contain P5, P8, and P10. However, some CLEs lack one or two Ps [6, 17]. Goad et al. concluded that proteins without a typical CLE domain were not CLEs, positing that the *Chlamydomonas reinhardtii* green algal genome did not contain CLE genes because it lacked the typical motif [18]. The putative candidate CLEs in *C. reinhardtii* contained a potential conserved CLE motif, ALVPSGPERRHH, but it lacks the typical histidine (H1) or arginine (R1) at the first amino acid position [18]. Sawa et al. previously reported the chemical structure of two *Arabidopsis* CLE peptides in vivo, *Arabidopsis* Tracheary elements (TEs) differentiation inhibitory factor (TDIF) and CLV3 [17, 19]. The mature *Arabidopsis* TDIF peptide is a dodecapeptide, and the sequence was highly homologous to part of the CLE domain. The CLV3 peptide had two prolines that were hydroxylated, similar to TDIF [17]. Ohyama et al. reported that CLV3 is a 13-amino-acid arabinosylated glycopeptide [14].

CLEs, as short-range signaling molecules, are a class of plant peptides that control stem cell fate [6]. CLE genes have also been shown to participate in various biological processes, such as plant growth, development, and responses to environmental stimuli [20]. CLE family genes can be divided into two types: A- and B-types. *Arabidopsis CLE41*, *CLE44*, and *CLE46* are B-type genes, while A-type genes include *CLV3*. A-type CLE peptides promote cell differentiation in root and shoot apical meristem [21]. The B-type CLE peptide *Arabidopsis* CLE41/TDIF/CLE44 promotes proliferation of vascular cells, while delaying differentiation into phloem and xylem cell lineages and regulating vascular stem cells [21, 22]. Similarly, *Arabidopsis CLE41* inhibits the differentiation of tracheary elements in *Zinnia elegans* [21]. *Arabidopsis CLE45* inhibits protophloem development and regulates pollen–pistil interactions under high temperature conditions [23–25]. The A-type CLE peptides of *Arabidopsis* (*CLV3*, *CLE19*, and *CLE40*) trigger consumption of the root meristem [26]. Overexpression of *CLE6* results in a short-root phenotype through inhibition of the root apical meristem (RAM) [21]. *Arabidopsis CLV3* was also shown to play roles in regulating development of stem cell niches of SAMs [12].

The mechanism by which CLE peptides regulate plant development remians unclear. CLEs primarily interact with LEUCINE-RICH REPEAT RECEPTOR-LIKE KINASEs (LRR-RLKs) [10, 27, 28] . In addition, CLEs regulate WUSCHEL-related homeobox proteins (WOXs) and that the CLE-RLK-WOX signaling system can regulate and maintain the meristem [12]. For example, *Arabidopsis* CLV3 signaling through these receptor complexes represses the transcription of WUS in the organizing center of the SAM, restricting stem cell divisions [29]. *Arabidopsis* CLE8 positively regulates *Arabidopsis* WOX8 either in the endosperm or in suspensor cells [12, 30]. *Arabidopsis* CLE40 may control *WOX5* expression in the RAM through interactions with the RLK ARABIDOPSIS CRINKLY4 [31].

Plant CLE peptides are also related to hormones. For example, *Arabidopsis* CLE41/TDIF interacts with brassinosteroids to determine xylem vessel formation by regulating GSK3s activity [22]. *Arabidopsis* CLE6 peptide can counter gibberellic acid (GA) deficiency to promote shoot growth [32]. TDIF/CLE41/CLE44 can interact with the LRR-RLK TDIF RECEPTOR (TDR)/PHLOEM INTERCALATED WITH XYLEM (PXY) expressed in the procambium and cambium [28, 33, 34]. TDR/PXY peptides are required for the auxin-dependent stimulation of cambium activity [35]. ETHYLENE RESPONSE FACTORs (ERFs) are required for normal vascular cell divisions, and in the absence of *TDR/PXY* and *WOX4* genes, expression of several ERFs are induced, suggesting that there is an interaction between TDIF/CLE41/CLE44-TDR/PXY-WOX4 signaling and ethylene signaling [36]. In addition, several CLE peptides are controlled by N-, P-, and S-responsive pathways in various ways [1].

However, CLE family members from many other species have not yet been identified and thus the functions of CLE family members from many species remain unknown. In 2013, the *CLV3-like* genes *VviCLE6*, *VviCLE25*, *VviCLE1*, and *TDIF* were identified from the grapevine by Tominaga-Wada et al. [37]. Later, Goad et al. identified the grape *CLE* genes *GSVIVT01024996001*, *GSVIVT01028623001*, *GSVIVT01020127001*, and *GSVIVT01016568001* [18]. However, many grape *CLE* genes remain to be identified, and the roles of grape *CLE* genes in most tissues and under various abiotic stresses remain unclear. In this study, we conducted a comprehensive analysis of grape *CLE* genes using the newly released grape genome (Version 2.1), and we investigated their expression profiles in different tissues and under different abiotic stresses. We identified nine putative *CLE* genes from the grape genome using a bioinformatics approach. The gene expression profiles in different tissues, codon usage bias and cis-regulatory elements of grape *CLE* genes were also analyzed.

## Results

### Identification of grape CLEs

BLASTP analyses were performed using the previously reported *Arabidopsis thaliana* CLE proteins and a conserved CLE motif (KRXVPXGPNPLHNR) as queries against proteins in the newest grape genome database (http://genomes.cribi.unipd.it/grape/). Previous reports have shown that CLE precursors are less than 300 aa in length [1], so candidates exceeding 300 aa in length were removed.

**Fig. 1:**
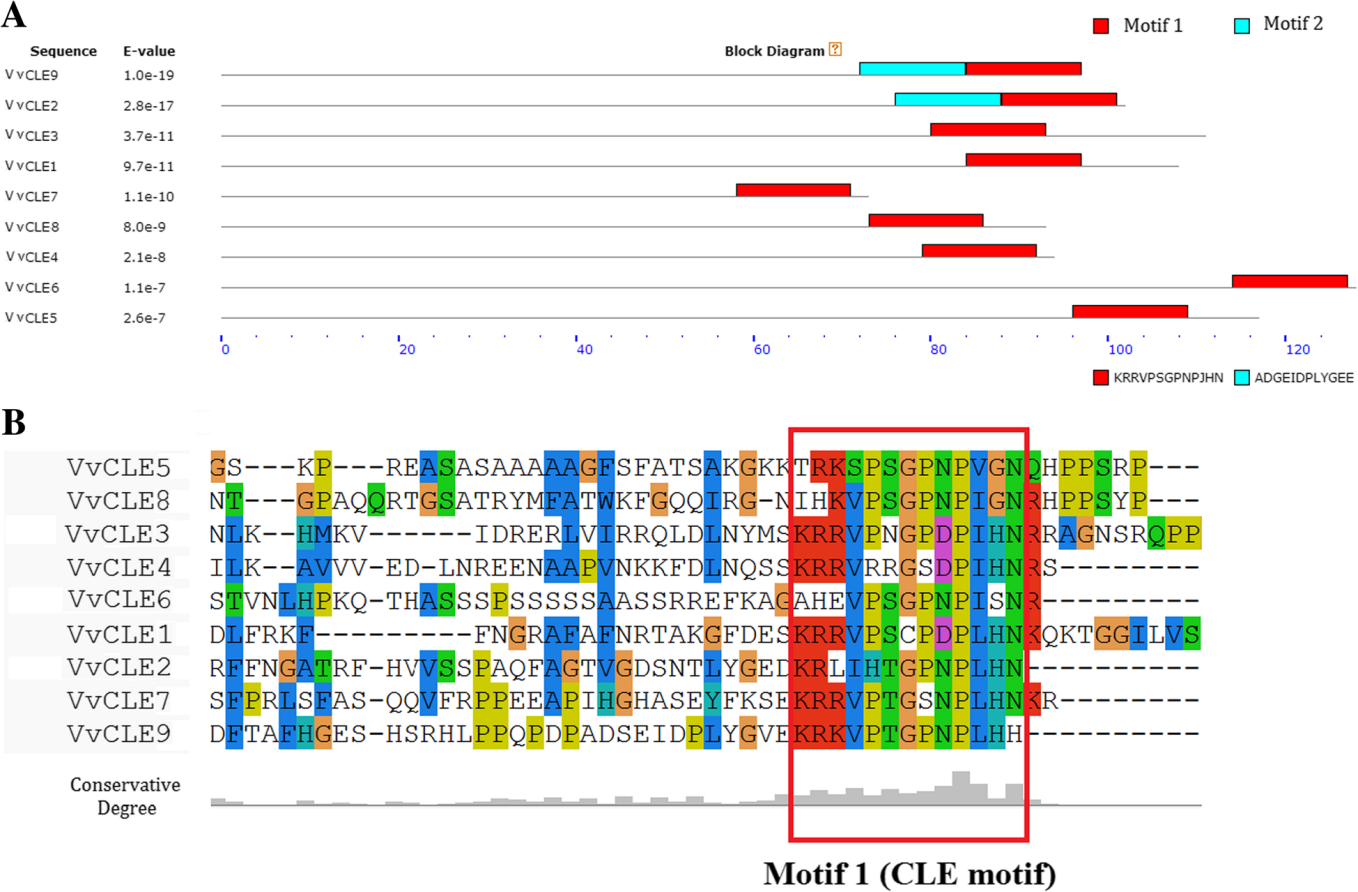
Motifs identified by MEME software and CLE motifs of grape CLEs. **a** represents the motifs identified by MEME. The red box contains motif 1 (CLE motif), and the blue box contains motif 2. **b** represents the alignment of grape CLE motifs, and the CLE motifs of grape CLEs are shown in the red box

The retrieved candidate genes were then filtered to identify proteins with a conserved C-terminal CLE motif [3]. To clearly identify the CLE motif, we considered the above factors in a typical CLE motif. We used the MEME software package (http://meme-suite.org/) to identify the conserved CLE motifs from these grape candidate CLEs. The following conserved motif parameters were used: motif length of 12–14 aa and output of two motifs, because past studies have shown that CLEs contain two CLE motifs [38]. Based on these parameters, we found the grape conserved CLE motif motif1 in *CLE* genes (Fig. 1). The conserved sequence of motif1 is KRRVPTGPBPLHN. Its length is 13 aa, and it typically contains R2, P5, P8, and P10. The conserved sequence of motif1 was inferred to be a typical CLE motif. Each grape candidate CLE that contained motif1 with an E-value < 0.01 was determined to have a CLE motif and categorized as a CLE. We used MEME to identify the conserved CLE motifs using the reported *Arabidopsis thaliana* CLEs and found the conserved motif KRLVPSGPBPLHN, which is the same as the previous reported motif sequence conserved in *Arabidopsis thaliana* (Han et al., 2016). Based on these strict conditions, we identified nine grape CLE genes (Table 1; E < 10^− 6^). We named the identified genes as *VvCLE1* to *VvCLE9* according to their order in the *V. vinifera* genomic sequence (Table 1).

**Table 1:** Grape CLEs that were identified

Gene ID	Gene name
*VIT_201s0011g03550*	*VvCLE1*
*VIT_201s0026g01090*	*VvCLE3*
*VIT_207s0005g06160*	*VvCLE6*
*VIT_213s0019g00920*	*VvCLE8*
*VIT_206s0004g04450*	*VvCLE5*
*VIT_217s0000g04203*	*VvCLE9*
*VIT_201s0011g05035*	*VvCLE2*
*VIT_212s0059g01135*	*VvCLE7*
*VIT_201s0026g02465*	*VvCLE4*

Our finding included all four *CLE* genes reported by Goad et al. [18]. Here, *GSVIVT01016568001* was named *VvCLE8*, *GSVIVT01024996001* was named *VvCLE5*, *GSVIVT01028623001* was named *VvCLE6*, and *GSVIVT01020127001* was named *VvCLE3*. Tominaga-Wada et al. identified grape *CLE* genes *VvCLE6*, *VvCLE25*, *VvCLE1*, and *TDIF*. Here, *VvCLE25* was named *VvCLE3*, and *VvCLE1* was named *VvCLE1* [36]. As “*VviCLE6”* identified by Tominaga-Wada et al. exceeded 300 aa in length, it was filtered out. TDIF did not contain a typical CLE motif, and it was also excluded.

### Analysis of gene expression and cis-regulatory elements

**Fig. 2:**
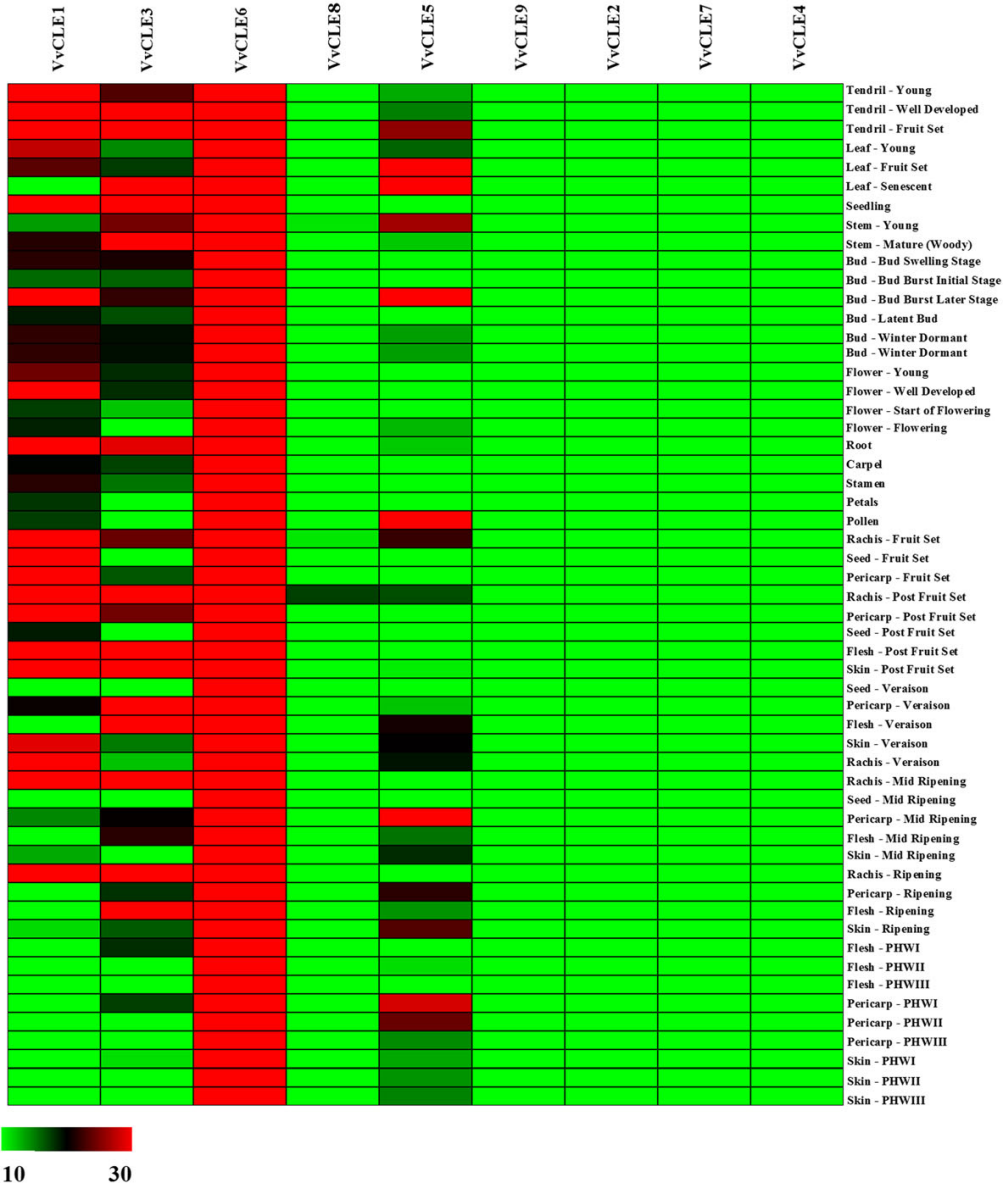
Heatmaps representing the expression profiles of grape *CLE* genes in 49 tissues The average values of RMA-normalized signal intensities of grape *CLE* genes were used to represent the expression level. High and low expression levels are shown in red and green, respectively

We analyzed the expression profiles of grape *CLE* genes in different tissues based on the microarray expression profiles of 49 grape samples (GSE36128) [39]. Expression levels were represented as the RMA-normalized signal intensity values of grape *CLE* genes. *VvCLE1* was expressed at a higher level in Tendril-Young, Seed-Fruit, Pericarp-Fruit, and Skin-Post Fruit sets and its highest expression was found in the Seed-Fruit set. Among these tissues, the signal intensity value of *VvCLE1* was > 50. *VvCLE3* was expressed at a higher level in the Tendril-Fruit, Stem-Mature (Woody), and Rachis-Ripening sets and the highest level in the Tendril-Fruit set. Among these tissues, the signal intensity value of *VvCLE1* was > 50. In most tissues, the signal intensity value of *VvCLE6* was > 50. In the Pericarp-Post Fruit, Flesh-Post Fruit Set, and Skin-Post Fruit sets, the signal intensity values for *VvCLE6* were > 1000 and its expression was highest in the Flesh-Post Fruit set. *VvCLE5* was expressed at the highest level at the Bud-Bud Burst Later stage, where it was expressed at > 50. In most tissues, the signal intensity value of *VvCLE8* was < 20, and its expression was highest in the Rachis-Post Fruit set (Fig. 2).

CLEs play roles as short-range signals to control the fate of stem cells [6]. Some WOXs can be regulated by CLEs, and the CLE-RLK-WOX signaling system can regulate and maintain the meristem [12]. Accordingly, we performed qRT-PCR to analyze the expression profiles of grape *CLE* genes in SAM-enriched regions, as well as shoot tissue without SAMs. *VvCLE3* was expressed in the SAM-enriched regions and not expressed in the shoot tissue without SAMs. *VvCLE9*, VvCLE2, *VvCLE8*, *VvCLE1,* and *VvCLE6* were not expressed in SAM-enriched regions, but were expressed in the shoot tissue without SAMs. Other *CLEs* were not expressed in both SAM-enriched regions and shoot tissues without SAMs (Additional file 3: Figure S1).

**Fig. 3:**
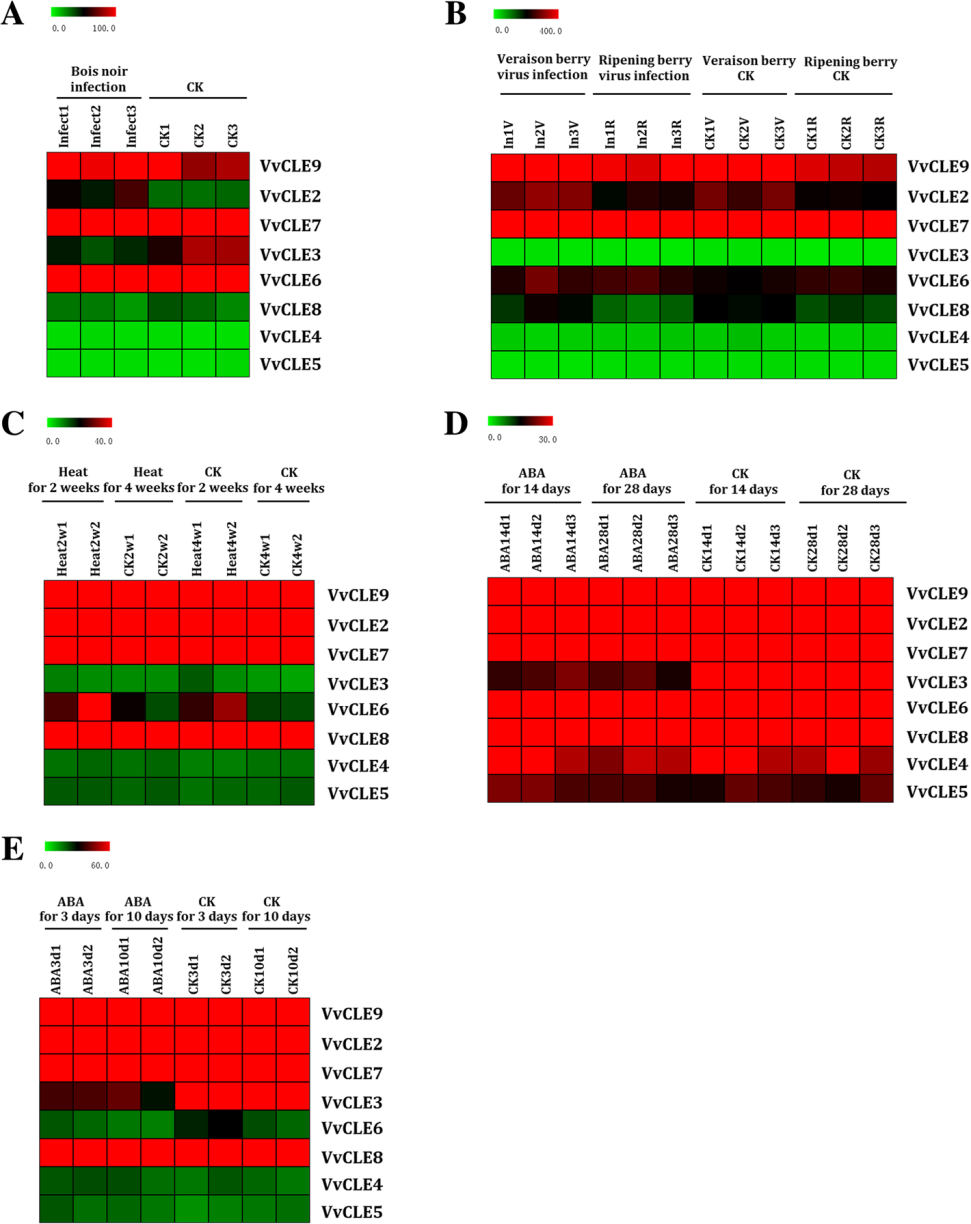
Heatmaps representing the expression profiles of grape *CLE* genes under biotic and abiotic stresses The average values of RMA-normalized signal intensities of grape *CLE* genes were used to represent the expression level. **a** represents the expression levels of grape *CLE* genes under bois noir infection. **b** represents the expression levels of grape *CLE* genes under GLRaV-3 virus infection. **c** represents the expression levels of grape *CLE* genes under high-temperature stress. **d** and **e** represent the expression levels of grape *CLE* genes under ABA treatment. Red and green denote high and low expression levels, respectively

Previous studies have shown CLE peptides can respond to external stimuli [20]. Accordingly, we analyzed the expression profiles of the grape *CLE* genes under exogenous abscisic acid (ABA) treatment, high-temperature stress, pathogenic fungi infection, and viral infection based on microarray expression data from the Plexdb database (http://www.plexdb.org/). Plants that were infected with bois noir (data from GSE12842) showed up-regulation of *VvCLE2* by 1.8-fold (*P* < 0.05; Fig. 3a). The expression patterns of *VvCLE* genes were not significantly affected in grapes infected with the GLRaV-3 virus (data from GSE31660). However, under GLRaV-3 virus infection and without GLRaV-3 virus infection (data from GSE31660), *VvCLE2* and *VvCLE9* were down-regulated (> 2-fold; *P* < 0.05), and *VvCLE7* was up-regulated (> 1.5-fold; *P* < 0.05) from veraison to berry ripening (Fig. 3b). *VvCLE6* was up-regulated by 1.9-fold (*P* < 0.05) in plants that were exposed to 4 weeks of high-temperature stress (data from GSE31675; Fig. 3c). Both in berry skins grown on the vine under ABA treatment and cultured in a Petri dishes under ABA treatment (GSE31664 and GSE31662), *VvCLE3* was down-regulated by > 2 fold (*P* < 0.05; Fig. 3d and e).

In addition, we analyzed the cis-regulatory elements of grape *CLE* promoters. Promoters of some A-type *CLE* genes, such as *VvCLE1*, *VvCLE2*, and *VvCLE3*, contained CAT-box elements, which are cis-acting regulatory elements involved in meristem expression [40]. Among the B-type *CLEs,* only the promoter of *VvCLE6* contained dOCT, a cis-acting regulatory element involved in meristem-specific activation [41]. Most grape *CLE* promoters contained the GCN4 motif or Skn-1 motif, which are cis-regulatory elements involved in endosperm expression [42, 43]. The promoters of some A-type grape *CLE* genes, such as *VvCLE1*, *VvCLE2*, and *VvCLE9*, contained ABRE, which is involved in responses to ABA [44]. However, the *VvCLE3* promoter, which has been shown to respond to ABA treatment in grape berry skin, did not contain ABRE. All B-type grape *CLE* promoters did not contain ABRE. Some grape *CLE* promoters contained GA-related cis-regulatory elements, such as the GARE motif [45]. *VvCLE1*, *VvCLE9*, and *VvCLE6* contained an as-2-box element, which is involved in shoot-specific expression [46]. Most grape *CLE* promoters contained HSE sequences, and some contained MeJA-responsiveness and salicylic acid responsiveness elements. *VvCLE1* and *VvCLE6* contained LTR elements, which are involved in cold stress responses [47]. *VvCLE9*, *VvCLE8*, and *VvCLE6* contained ERE elements, which are ethylene responsive [48]. *VvCLE9*, *VvCLE2*, and *VvCLE8* contained O2 sites, which are cis-acting regulatory elements involved in zein metabolism regulation [49] (Additional file 2: Table S2).

### CLE comparison between grape and other species

**Fig. 4:**
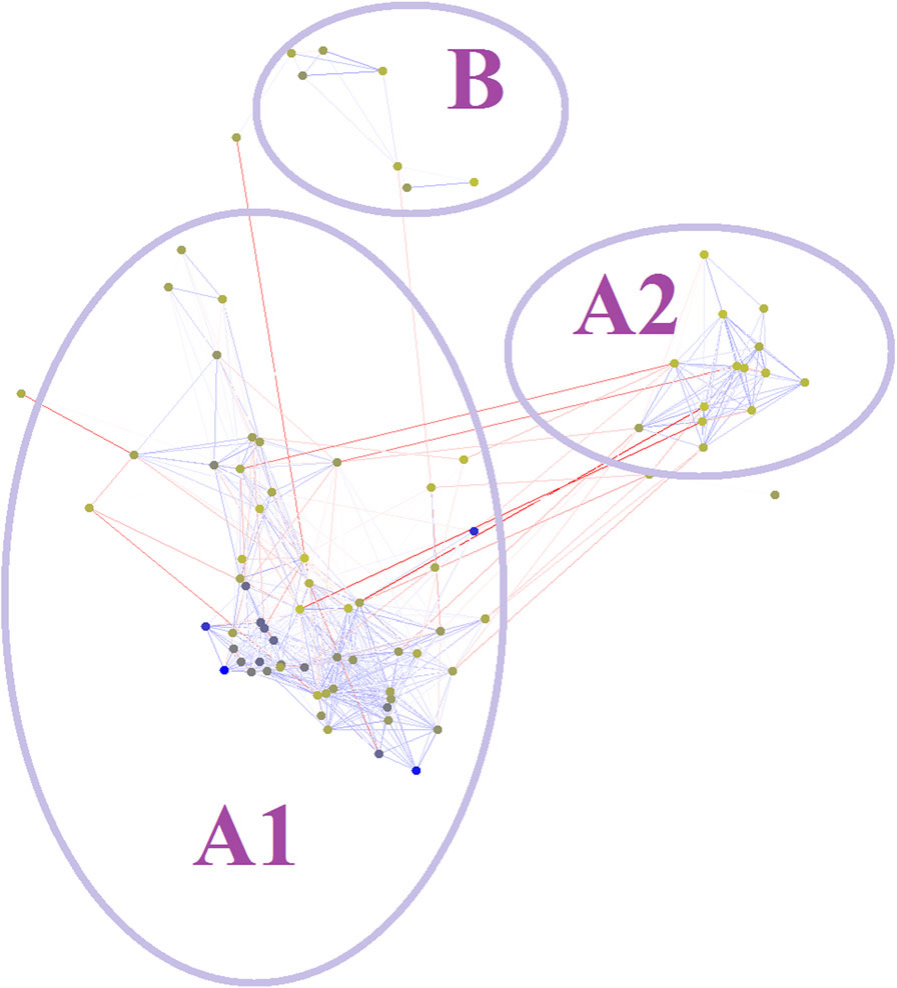
Clustering diagram of CLEs. Blue lines connect CLEs in the same cluster and circles represent the CLEs. Blue circles represent long-length proteins and yellow circles represent short-length proteins. Clusters A1, A2, and B represent the CLE genes clustered into groups A1, A2, and B, respectively

**Fig. 5:**
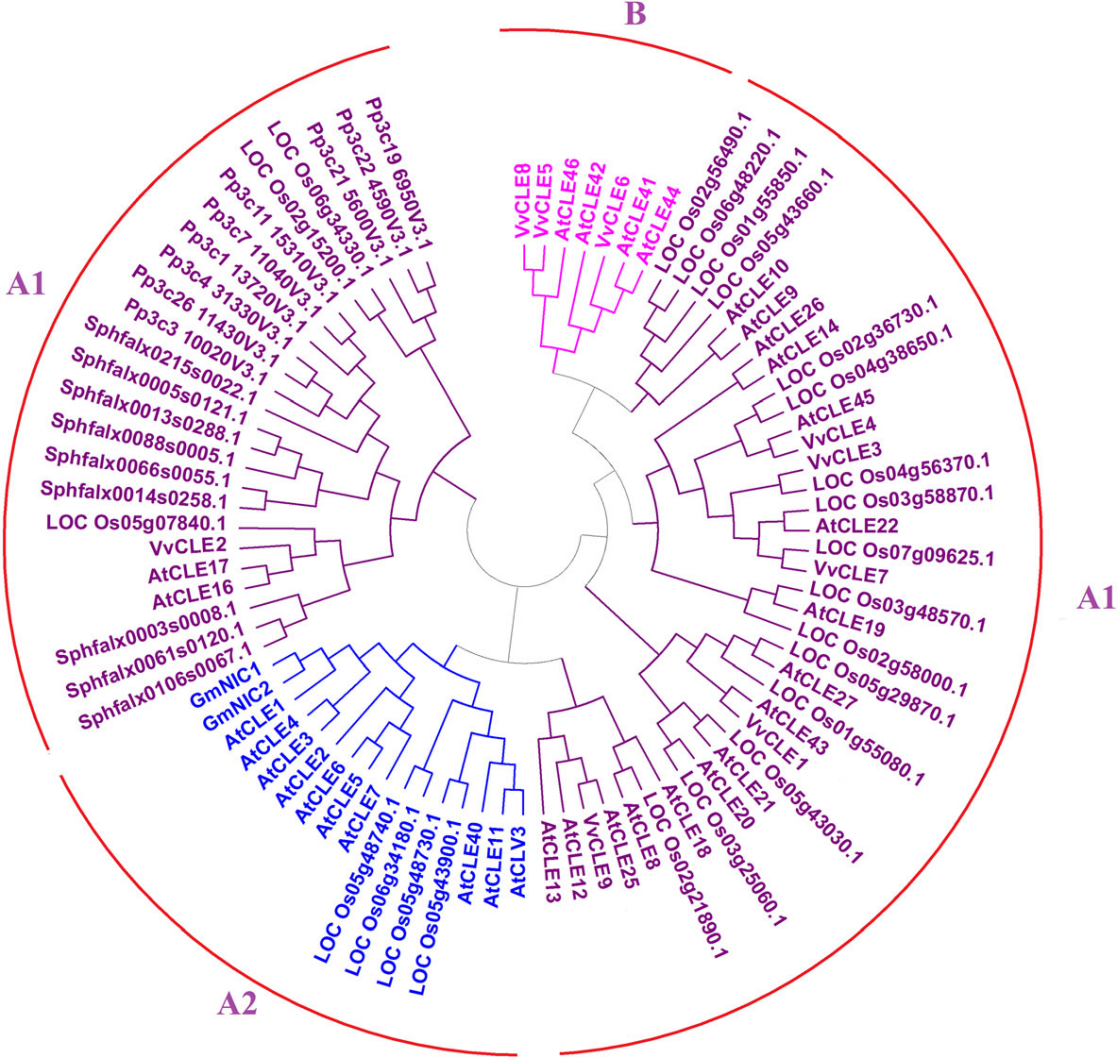
Neighbor-joining tree analysis of CLEs Branches A1, A2, and B represent groups A1, A2, and B, respectively. Gene names indicated in purple belong to A1 group, blue gene names belong to A2 group and purple-red gene names belong to B group

We compared the characteristics of grape CLEs with CLEs in other species, including the non-angiosperms *Physcomitrella patens* and *Sphagnum fallax*, the monocotyledon rice, and the dicotyledons *Arabidopsis thaliana* and two soybean CLEs (*GmNIC1* and *GmNIC2*) that are regulated by nodulation [18, 36, 50]. We structured the clustering diagram of the above CLEs using the CLANS, which can create two-dimensional clustering diagrams. CLEs from different species were divided into three groups: A1, A2, and B (Fig. 4). The B group included *AtCLE41*, *AtCLE42*, *AtCLE44*, *AtCLE46*, *VvCLE6*, *VvCLE8*, and *VvCLE5*, which are all B-type CLEs that were included in this study. The A1 and A2 groups contained all the A-type CLEs that were included in this study. We used MEGA to generate a phylogenetic tree of the CLEs. The classification based on the phylogenetic analysis was similar to the classification determined by CLANS. The phylogenetic tree showed that CLEs could be divided into four clusters. One cluster included only the B group members identified by CLANS, and we named it the B cluster. One cluster included only the A2 group members identified by CLANS, and we named it the A2 cluster. The A2 group/A2 cluster contained *GmNIC1* and *GmNIC2*, which are regulated by nodulation [18, 36, 50]. Goad et al. concluded the group containing *GmNIC1* and *GmNIC2* may be involved in mycorrhizal development, as mycorrhizae and nodulation are thought to share overlapping gene regulatory networks [18]. However, the A2 group did not contain any grape, rice, or moss *CLE* genes. The other two clusters identified by phylogenetic analysis included only the A1 group members identified by CLANS. Phylogenetic analysis showed that the two A1 clusters could be divided into ancestral and modern clusters. The ancestral cluster contained *P. patens* and *S. fallax* CLEs, though the modern cluster did not. We found the non-angiosperms, including *P. patens* and *S. fallax*, did not have B group, B-type members or A2 group members. Phylogenetic analysis showed that rice may not contain B-type members (Fig. 5).

**Fig. 6:**
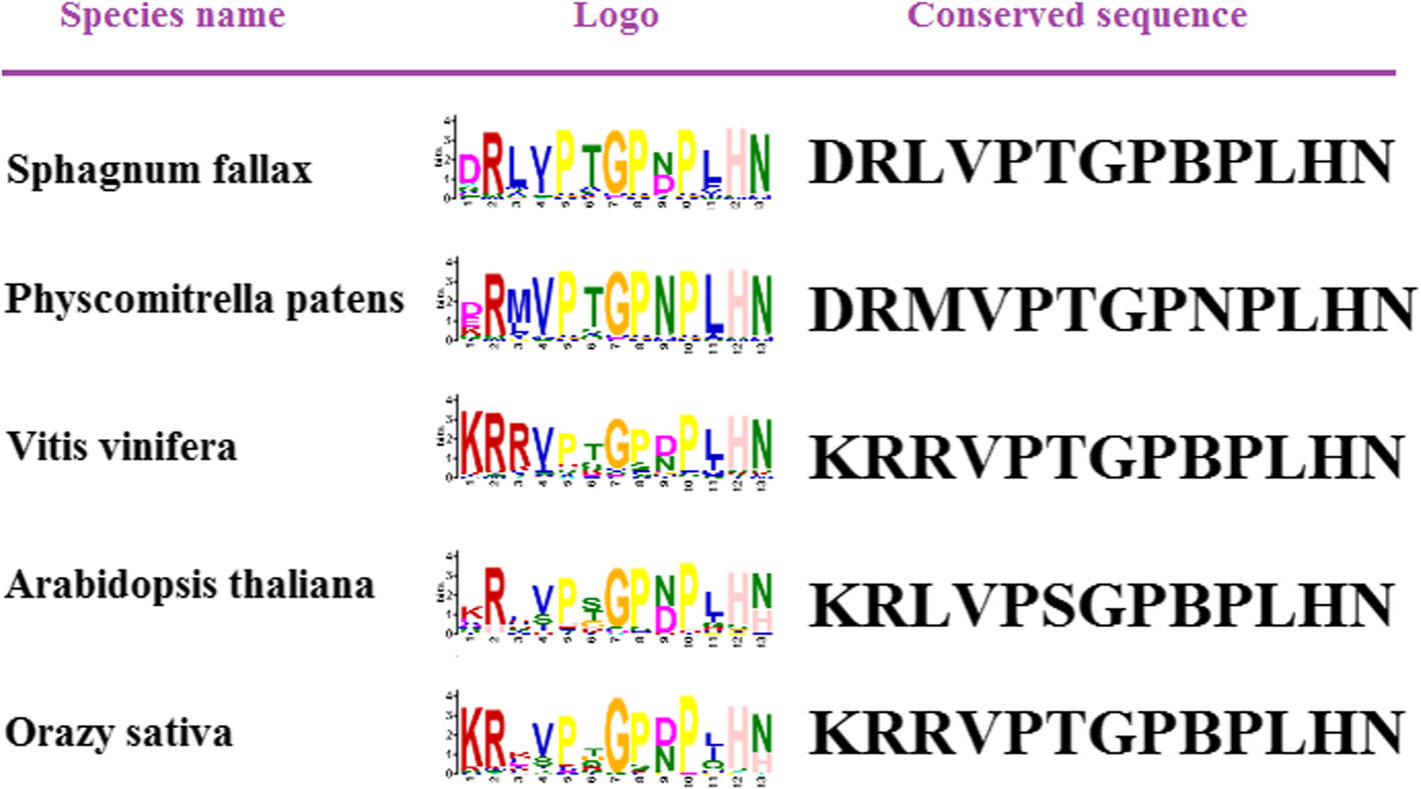
LOGO and conserved sequences of CLEs in different species. Species name is indicated on the left. The conserved LOGO of CLE sequences of different species is indicated in the middle. The right denotes the conserved sequences of CLEs from different species

We compared the conserved sequences of grape CLE motifs with the CLE motif in other species. We identified the conserved sequence of grape CLE motifs and the CLE motif in other species using the MEME software package as described above. We found that the conserved CLE motif sequences of the non-angiosperms *P. patens* and *S. fallax* contained D1, while the other species contained K1. The conserved C-terminal motif of *P. patens* was NPLHN, while the C-terminal motif in other species was BPLHN (Fig. 6). In some *Physcomitrella patens*, *Arabidopsis thaliana*, and rice CLEs, a 12-aa-long conserved motif sequence was identified using MEME software with the above parameters (where the motif length is 12–14 aa and the output is two conserved motifs). These conserved motifs contained a “LLLL” sequence (Additional file 4: Figure S2). In *A. thaliana*, we found *AtCLE8* and *AtCLE22* contained two CLE motifs. In rice, we found *LOC_Os05g29870* contained six repeat CLE motifs (Additional file 5: Figure S3).

The ratio of nonsynonymous/synonymous substitution rates (*K*_a_/*K*_s_) can provide a measure of selective pressure. *K*_a_/*K*_s_ values of 1, < 1, and > 1 indicate neutral evolution, purifying selection, and positive selection, respectively [51, 52]. *K*_a_/*K*_s_ analysis showed that only the *S. fallax* CLE family underwent positive selection, while the CLE families in other species underwent purifying selection (Table 2) [51, 52]. The *K*_a_/*K*_s_ value of grape CLE genes was 0.7, suggesting that these genes underwent weak purifying selection. However, the *K*_a_/*K*_s_ value of grape CLE motifs was 0.03, and no positive selection sites were found, indicating that the grape CLE motifs underwent strong purifying selection.

**Table 2:** Selection pressure analysis of CLE families in different species

Specie name	Ka/Ks value of CLE family	Positively selected site number
*Physcomitrella patens*	0.83	5
*Sphagnum fallax*	1.17	134
*Arabidopsis thaliana*	0.52	2
*Vitis vinifera*	0.7	22
*Orazy sativa*	0.82	52

**Fig. 7:**
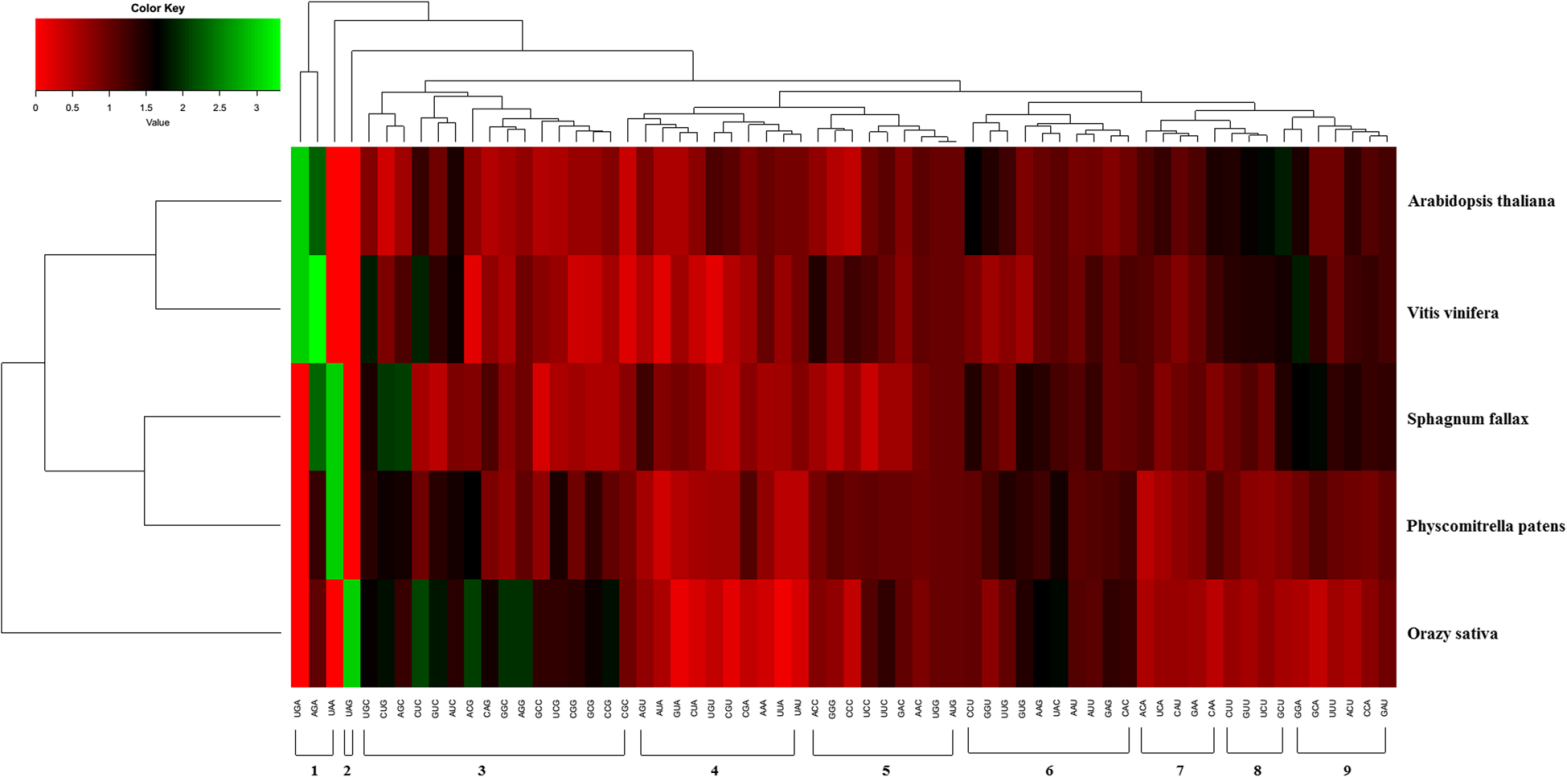
Heatmaps representing the RSCU values of CLE families in different species 1–9 represent each of the nine codon groups

The average GC and GC3s content of rice *CLE* genes were highest compared to the other species’ *CLE* genes. The average CAI and Fop were highest in rice *CLE* genes, and the average effective number of codons (ENCs) was lowest in rice *CLE* genes compared with the CLE genes of other species. This indicated that the codon usage bias of the rice CLE gene family was strongest (Table 3). In *P. patens* and *Arabidopsis thaliana*, the *CLE* genes’ CAI and Fop values were not correlated with GC and GC3s content. In rice, the *CLE* genes’ CAI and Fop values were positively correlated with GC and GC3scontent (*r* > 0.7; *P* < 0.05). In grape, the CLE genes’ CAI and Fop values were positively correlated with GC and GC3s content (*r* > 0.6; *P* < 0.05). In *Sphagnum fallax*, the CLE genes’ CAI and Fop values were positively correlated with GC content (*r* > 0.6; *P* < 0.05) and were not correlated with GC3s content. Relative synonymous codon usage (RSCU) is the observed frequency of a codon divided by the expected frequency. RSCU < 1 indicates less-used codons, and RSCU > 1 indicates that the codons are used more frequently than expected [53]. Non-angiosperms *P. patens* and *S. fallax* had similar RSCU values, while the RSCU values were similar for Dicotyledon *Arabidopsis thaliana* and grape *CLE* families. Both in *P. patens* and *S. fallax* CLE families, the RSCU of UAA and AGA codons were higher. Both in *Arabidopsis thaliana* and grape CLE families, the RSCU of UGA and AGA codons were higher. In the rice CLE family, the RSCU of UAG, CUC, ACG, GGC, and AGG codons were higher. For these five species, we found that CLE family codons could be classified into nine groups based on their RSCU values (Fig. 7). In the same group, the RSCU of codons tended to be consistent among species. We found that codons in same group end with the same base type except for group 6 (i.e., AT type/GC type; Fig. 7).

**Table 3:** Codon preference index of CLE families in different species

Specie name	CAI	CBI	Fop	ENC	GC3s	GC content
*Vitis vinifera*	0.176	−0.007	0.406	50.706	0.462	0.484
*Arabidopsis thaliana*	0.177	0.000	0.403	50.371	0.406	0.448
*Orazy sativa*	0.186	0.081	0.454	40.705	0.715	0.650
*Physcomitrella patens*	0.161	0.001	0.396	55.021	0.573	0.561
*Sphagnum fallax*	0.184	−0.004	0.408	53.232	0.436	0.489

### Gene duplication and loss event analysis

Compared with *A. thaliana*, tomato, and *Populus trichocarpa*, grape had fewer *CLE* genes [3, 12, 13]. The previous study found that *CLE* genes in grape, *S. fallax* (nine CLE genes) and *P. patens* (nine *CLE* genes) were outnumbered relative to those in most species [18].

We used the Notung software package to identify gene duplication and loss events. By comparing grape and *P. patens*, Notung identified the duplication and loss events in both grape and *P. patens* (Additional file 6: Figure S4a). By comparing grape and *S. fallax*, Notung identified the duplication events in both grape and *Sphagnum fallax* but only identified loss events in *S. fallax* (Additional file 6: Figure S4b). By comparing grape and *A. thaliana*, Notung only identified loss events in grape (Additional file 6: Figure S4c).

## Discussion

Goad et al. [18] found four *CLE* genes in grape. Their study identified fewer *CLE* in various species than previously expected. For example, one previous study found 32 *Arabidopsis thaliana CLE* genes, while Goad et al. found 31. Another previous study found 44 soybean *CLE* genes, while Goad et al. found 43 [12, 18, 54]. Our method was able to find more reliable grape *CLE* genes at a genome-wide scale in grape.

In grape, fewer *CLE* genes were found compared with other species, such as *A. thaliana*, *P. trichocarpa*, and tomato [3, 12, 13]. *AtCLV3* was shown to play roles in regulating development of stem cell niches of SAMs [12]. Over-expression of *AtCLE6* in a GA-deficient mutant partially rescued the mutant phenotype, suggesting that CLE6 can compensate for GA deficiency to promote shoot growth in *Arabidopsis* [32] and may play a role in the GA pathway. *AtCLE6* is also associated with procambium proliferation [20]. We found grape *CLEs* similar to *AtCLE6* that may have implications for grape cultivation, such as elongating grape spikes and grape flowers, as well as fruit thinning. However, we did not find homologs of *AtCLV3* and *AtCLE6* in grape. We analyzed the expression profile of grape *CLE* genes and found that grape *CLE* genes likely play a role in shoot or SAM development. We found that grape *VvCLE3* was expressed in most tissues, including SAM tissue. In our study, other grape *CLE* genes were not expressed in SAM tissue. We also found that *VvCLE3* was down-regulated under exogenous ABA treatment. Previous studies have shown that some CLEs interact with hormones or are related to hormones. For example, AtCLE41/TDIF can interact with brassinosteroids (BR) [22], and AtCLE6 peptide can counter GA deficiency to promote shoot growth [32]. TDR/PXY are required for the auxin-dependent stimulation of cambial activity [35]. ERFs are required for normal vascular cell divisions, and in the absence of *TDR/PXY* and *WOX4* genes, expression of several ERFs are induced, suggesting an interaction between TDIF/CLE41/CLE44-TDR/PXY-WOX4 signaling and ethylene signaling [55].

A previous study showed that the CLE45 peptide can mediate environmental signals [20]. CLE45 is preferentially expressed in the stigma at normal temperatures, whereas its expression domain expands into the transmitting tract at elevated temperatures, suggesting that CLE45 has temperature-dependent functions [24]. With the intensification of the greenhouse effect and global warming, plant responses to heat stress are particularly important. We found that only *VvCLE6* responded to heat stress, so we speculate that *VvCLE6* was the only *CLE* gene involved in heat stress responses in grape. Under bois noir or GLRaV-3 virus infections, most grape *CLE* genes did not exhibit significant changes compared with normal conditions.

We found that the A-type grape *CLE* genes *VvCLE1*, *VvCLE2*, and *VvCLE3* contained a CAT-box, which is a cis-acting regulatory element involved in meristem expression. However, *VvCLE1* and *VvCLE2* were not expressed in grape SAM, though they may be expressed in other meristem tissues. In *Arabidopsis*, CLE43 peptides suppress xylem differentiation [18, 21]. *CLE17* is expressed in the RAM, the lateral root cap cells, and the epidermis, and *CLE17* is involved in root development [56].

In B-type *CLE* genes, only *VvCLE6* contained dOCT, which is a cis-acting regulatory element related to meristem-specific activation. In *Arabidopsis*, CLE44 or CLE41 are involved in the regulation of vascular stem cells [22, 28, 34, 55]. *VvCLE1*, *VvCLE2*, and *VvCLE9* contained the ABRE element, while *VvCLE3* did not. However, only *VvCLE3* had altered expression under exogenous ABA treatment. B-type *CLE* genes did not contain ABRE. *VvCLE6* and some other grape *CLE* genes contained HSE. In addition, many grape *CLE* promoters contained some hormone responsiveness elements, indicating that they may be involved in hormone signal pathways, such as those of GA, jasmonic acid, salicylic acid, and ethylene (Additional file 2: Table S2).

We constructed a 2-D clustering diagram of all CLEs using the grape, *P. patens*, *S. fallax*, *A. thaliana*, and rice CLEs as well as two soybean CLEs. These CLEs were divided into three groups: A1, A2, and B. The phylogenetic analysis showed a similar pattern for the A2 and B groups. A2 was divided into two clusters, the ancestral and modern clusters. In the ancestral cluster, there were only non-angiosperms *P. patens* and *S. fallax CLE* genes, matching findings by Goad et al. [18], who found that *CLE* genes from non-angiosperms *P. patens* and *S. fallax* could be divided into an independent cluster [18]. Dicotyledon or monocotyledon CLEs could not be divided into an independent cluster, also consistent with the findings of Goad et al. [18]. The A2 group contained *GmNIC1* and *GmNIC2*, which are regulated by nodulation [18, 40, 41]. Goad et al. concluded that the group with *GmNIC1* and *GmNIC2* could be involved in nodulation or mycorrhizal development, as mycorrhizae and nodulation share overlapping gene regulatory networks [18]. However, the A2 group did not contain any grape, rice, or moss *CLE* genes. A previous study indicated that some *CLE* genes were related to nodulation and could modulate nodulation [57]. Nematodes may have also secreted nematode CLEs, and nematode CLEs function like endogenous plant CLE peptides. Once nematode CLEs are delivered into plant cells, they can function similarly to endogenous plant CLEs to redirect plant CLE signaling pathways to establish a successful parasitic association with host plants [58]. Grapes are also invaded by rhizobia, nematodes, and *Phylloxera*. While group A2 did not contain any grape *CLE* genes, we found that some grape *CLE* genes, such as *VvCLE1* and *VvCLE6,* were expressed at a higher level in grape root tissue. Furthermore, these CLE genes should be studied to better understand the relationship of grape plants with rhizobia, nematodes, and *Phylloxera*. Groups B and A2 did not contain any moss *CLE* genes. Because moss plants do not have true root or vascular tissues, moss CLEs cannot be exclusively involved in functions related to root tissue, vascular tissue, or nodulation. This may explain why groups B and A2 did not contain any moss *CLE* genes (Fig. 4).

We found *P. patens*, *A. thaliana,* and rice CLEs that contained a 12-aa-long motif. The most conserved part of the motif was “LLLL.” The function of the motif is unclear (Additional file 4: Figure S2). A previous study has shown some CLE genes contain two CLE motifs [38]. In rice, we found the *LOC_Os05g29870* contained six repeated CLE motifs. Another peculiarity of the rice CLE family was its strong codon bias compared with the other four species. Its average Fop and CAI were highest and its GC3s and GC content were highest as well. In contrast, the *S. fallax* CLE family was the only family that underwent positive selection. If codon usage bias is correlated with GC content, we could deduce whether codon usage bias was affected by mutation pressure during its evolutionary history [59–61]. The Fop and CAI values of rice and grape CLE family members were positively correlated with GC and GC3s content, suggesting that rice and grape *CLE* genes mainly evolved by mutation pressure. The Fop and CAI values of *P. patens* and *A. thaliana* CLE family members were not positively correlated with GC and GC3s content, indicting their *CLE* genes evolved by other pressure, such as natural pressure [59, 60].

We identified some gene duplication events in *CLE* genes. The analysis conducted with Notung identified gene duplication events in grape, *P. patens*, *S. fallax*, and *A. thaliana* therefore gene duplication may have contributed to the expansion of the CLE family. Both rice and *Arabidopsis thaliana* have undergone one γ events (ancient whole-genome replication events) and at least two whole-genome duplication (WGD) events, and grapes appear to have only undergone one γ event  [62]. *P. patens* appear to have experienced at least two WGD events [63], and *S. fallax* has undergone at least one WGD event [64]. More gene duplication and WGD events have been identified in *A. thaliana*. This result indicates that WGD may have played an important role in gene duplication events in the CLE family. The analysis with the Notung software indicated that gene loss events have occurred in grape and moss. Gene loss events or lower gene duplication rates may have led to grape and moss containing fewer *CLE* gene family members than *Arabidopsis thaliana*.

## Conclusions

The present study provides an effective method for identifying CLE motifs and increases the understanding of grape CLEs. Moreover, our systematic analysis provided comprehensive information for further research investigating the functions of grape CLEs. Future research on *CLE* genes may have applications for grape breeding and cultivation to better understand root and nodulation development.

## Methods

### Data collection and identification of grape CLE genes

Grape protein data was obtained from the grape genome database (http://genomes.cribi.unipd.it/grape/). CLEs that were previously identified in *Arabidopsis thaliana* and a CLE conserved motif (KRXVPXGPNPLHNR) were used as queries to perform BLASTP analysis (E < 10^− 20^). Candidate proteins without a conserved C-terminal CLE motif and with candidate protein lengths exceeding 300 aa were removed.

### Multiple sequence alignment, phylogenetic, and two-dimensional clustering analysis

Protein multiple sequence alignment was performed using MAFFT v7 (https://mafft.cbrc.jp/alignment/server/), and Neighbor-joining (NJ) trees were constructed using MEGA 6.0 based on full-length protein sequences [65]. To support the inferred relationships, 1000 bootstrap samples were generated. CLANS was used to construct the two-dimensional clustering diagram [66].

### Analysis of selective pressure

The selective pressures on sequences were determined using Codeml in PAML (phylogenetic analysis maximum likelihood) version 4.7 software [51].

### Gene duplication and loss event analysis

Notung 2.9 [67] was used to analyze gene duplication and loss events.

### Codon usage bias analysis

The frequency of optimal codons (FOP), GC content, GC content at the third site of synonymous codons (GC3s content), relative synonymous codon usage (RSCU) and codon adaptation index (CAI) were analyzed using the coding sequences of *CLE* genes from grape and other species with CodonW 1.4.2.

### Plant materials, and RNA isolation

SAM enrichment regions and shoot tissue samples without SAMs were collected from grapes cv. ‘Cabernet Sauvignon.’ RNA was isolated using the CTAB method as described previously [68]. DNase I was used to digest genomic DNA and to eliminate DNA contamination. Reverse transcription was also performed as previously described [69].

### Gene expression analysis

Quantitative real time PCR for grape *CLE* genes was performed as previously described [69]. The qRT-PCR primer sequences are provided in Additional file 1: Table S1. *VvActin7* (*VIT_204s0044g00580*) was used as the reference sequence [70]. The *VvActin7* forward and reverse primers were 5′-CTTGCATCCCTCAGCACCTT-3′ and 5′-TCCTGTGGACAATGGATGGA-3′, respectively [60]. Three biological replicates were included for each analysis, and each biological replicates were analyzed through three technical replicates. Gene relative expression levels were calculated using the ^ΔΔ^CT method.

The microarray expression profiles of bois noir–infected samples (GSE12842), GLRaV-3 virus–infected samples (GSE31660), high-temperature-treated samples (GSE31675), ABA-treated samples (GSE31664 and GSE31662), and 49 tissue samples (GSE36128) from grape plants were retrieved from the Plexdb (http://www.plexdb.org/) and GEO databases (https://www.ncbi.nlm.nih.gov/gds/). The RMA-normalized signal intensity values of grape *CLE* genes were used to represent the expression level. The average values of replicates were used to calculate the fold changes. *T-test p*-values < 0.05 were considered to be significantly different. The pheatmap R package was used to generate heatmaps.

### Analysis of cis-acting regulatory elements in promoters

We identified cis-acting regulatory elements of grape *CLE* promoters using Plantcare software (http://bioinformatics.psb.ugent.be/webtools/plantcare/html/), as previously described [69].

## Additional files

Additional file 1: **Table S1.** qRT-PCR primers for *CLE* genes in grape. (XLS 15 kb)

Additional file 2: **Table S2.** Cis-acting elements of *CLE* promoters in grape. (XLS 15 kb)

Additional file 3: **Figure S1.** Relative expression level of grape *CLE* genes in shoot apical meristem (SAM)-enriched regions and the shoot tissue without SAMs. (PNG 89 kb)

Additional file 4: **Figure S2.** LOGO and conserved sequences of the “LLLL” motif in CLEs from three species. (PNG 27 kb)

Additional file 5: **Figure S3.** Motifs identified from *Arabidopsis* and rice CLEs by MEME A and B represents the motifs identified by MEME in *Arabidopsis thaliana* and rice, respectively. The red box contains motif 1 (CLE motif), and the blue box contains motif 2 (LLLL motif). (PNG 134 kb)

Additional file 6: **Figure S4.** Gene duplication and loss events in CLE families based on Notung analysis. A, B, and C represent the duplication and loss events of *Physcomitrella patens*, *Sphagnum fallax*, and *Arabidopsis thaliana* relative to grape, respectively. D (in red) represents duplication events and “LOST” (in gray) represents loss events. (PNG 142 kb)

## Data Availability

The data sets supporting the results of this article are included within the article and its additional files.

## Abbreviations

CEP: C-TERMINALLY ENCODED PEPTIDE
CLE: CLAVATA3/EMBRYO SURROUNDING REGION
ERF: ETHYLENE RESPONSE FACTOR
LRR-RLK: LEUCINE-RICH REPEAT RECEPTOR-LIKE KINASE
PXY: PHLOEM INTERCALATED WITH XYLEM
RAM: Root apical meristem
SAM: Shoot apical meristem
TDR: LRR-RLK TDIF RECEPTOR
TE: Tracheary element
