# Supplementary figures and images for: Genome-wide identification of the CLAVATA3/EMBRYO SURROUNDING REGION (CLE) family in grape (Vitis vinifera L.)
Source: BMC Genomics. 2019 Jul 5;20:553. doi: 10.1186/s12864-019-5944-2 (PMC6612224; doi:10.1186/s12864-019-5944-2)

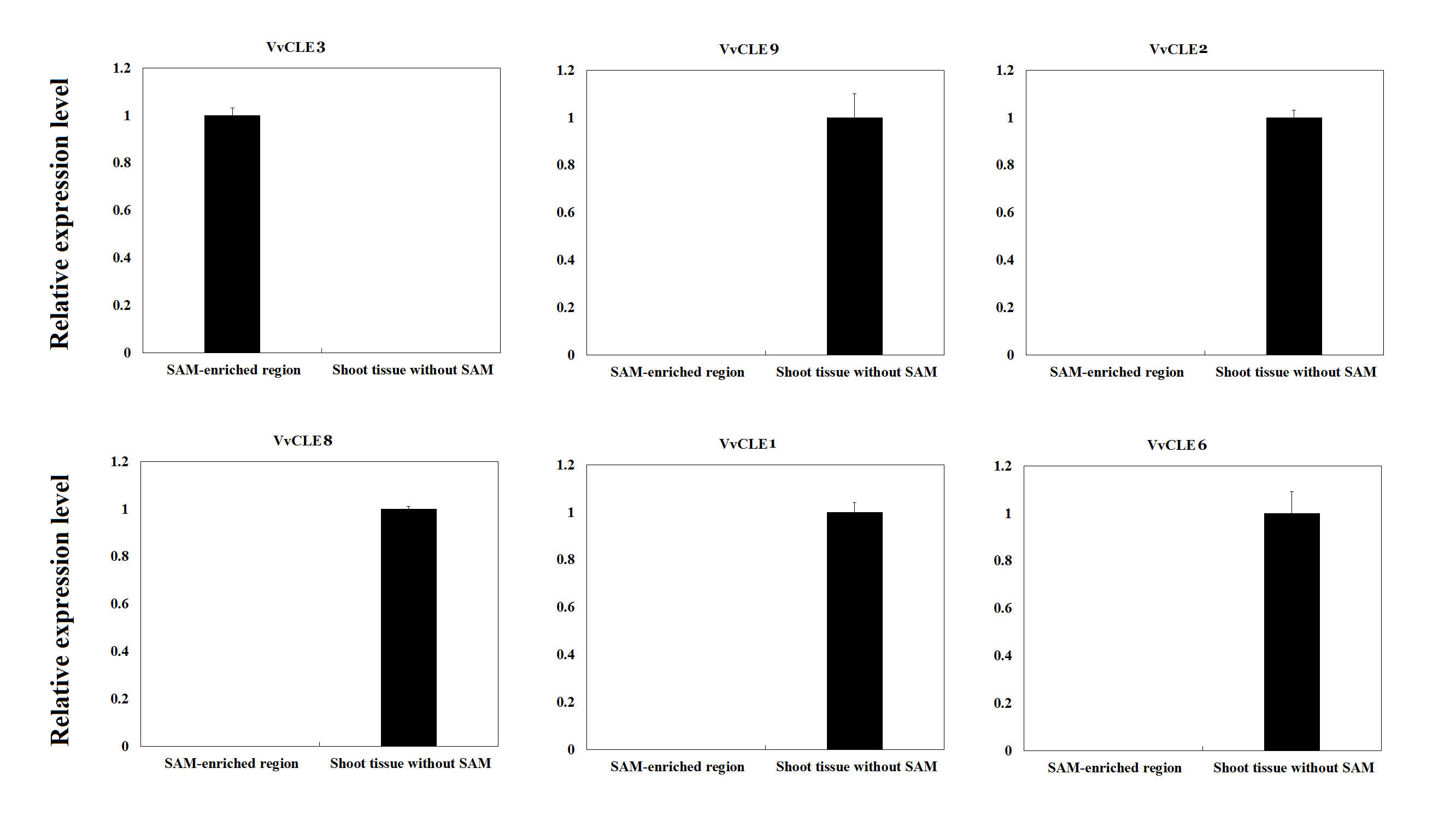

Supplement: Supplementary file 3 — Figure S1. Relative expression level of grape CLE genes in shoot apical meristem (SAM)-enriched regions and the shoot tissue without SAMs. (PNG 89 kb) [file 12864_2019_5944_MOESM3_ESM.png]

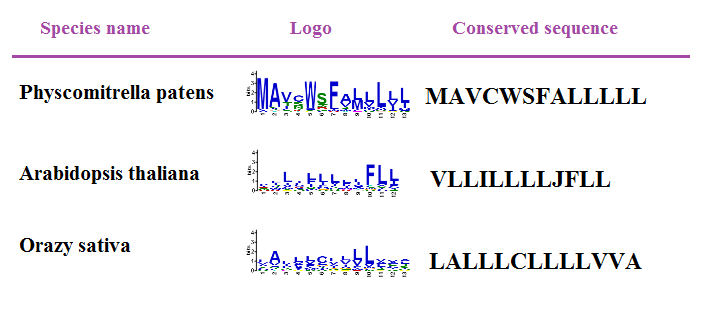

Supplement: Supplementary file 4 — Figure S2. LOGO and conserved sequences of the “LLLL” motif in CLEs from three species. (PNG 27 kb) [file 12864_2019_5944_MOESM4_ESM.png]

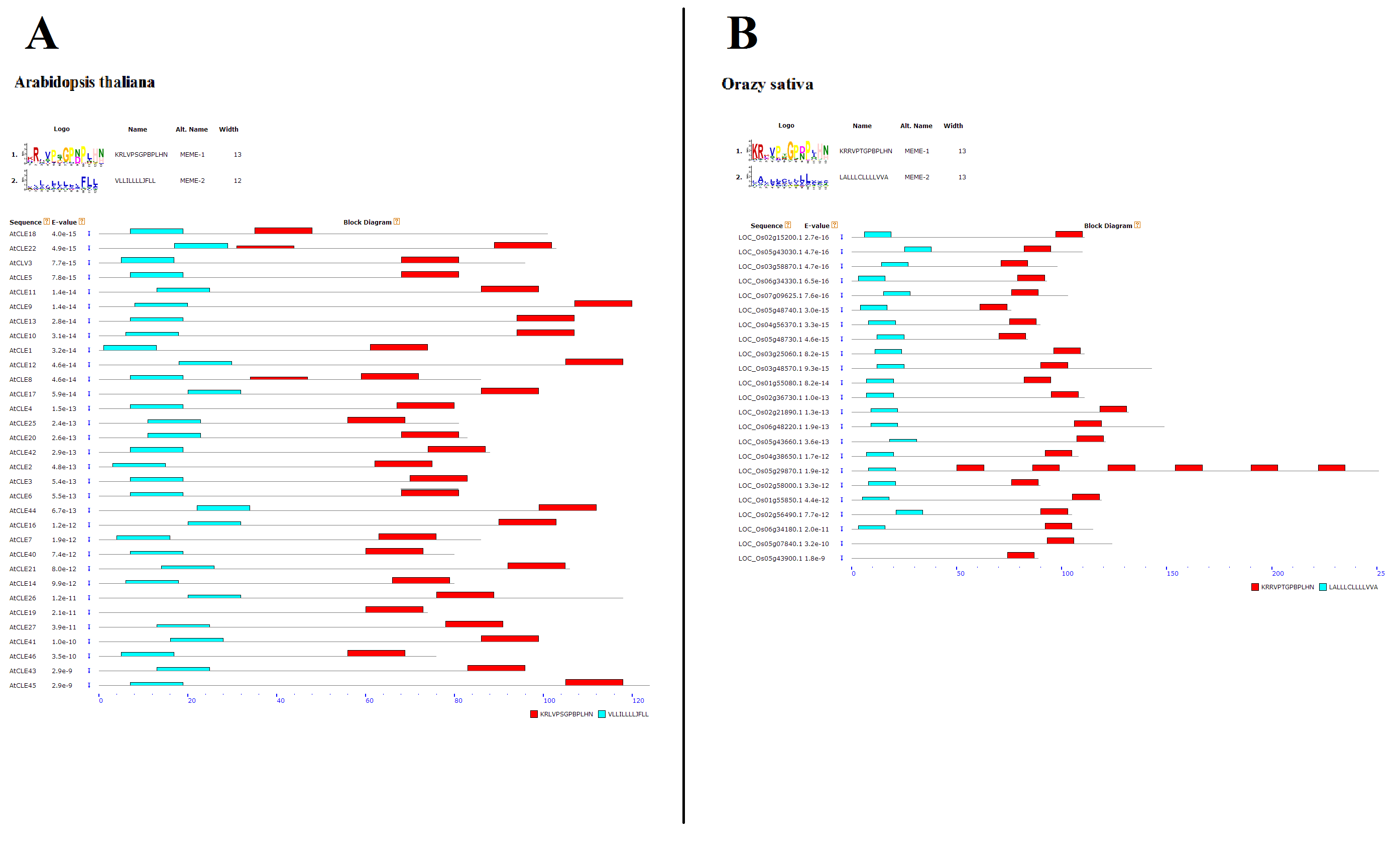

Supplement: Supplementary file 5 — Figure S3. Motifs identified from Arabidopsis and rice CLEs by MEME A and B represents the motifs identified by MEME in Arabidopsis thaliana and rice, respectively. The red box contains motif 1 (CLE motif), and the blue box contains motif 2 (LLLL motif). (PNG 134 kb) [file 12864_2019_5944_MOESM5_ESM.png]

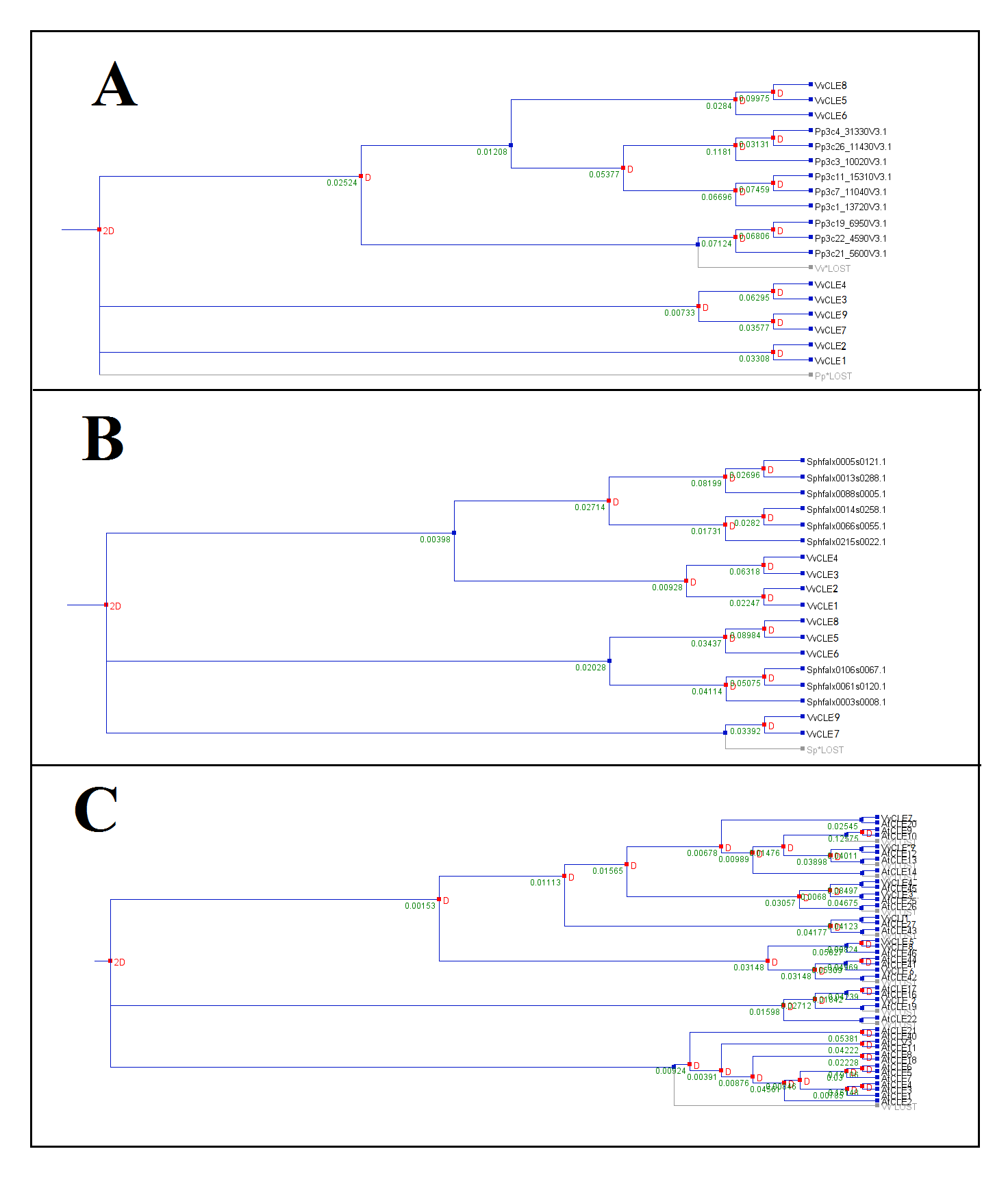

Supplement: Supplementary file 6 — Figure S4. Gene duplication and loss events in CLE families based on Notung analysis. A, B, and C represent the duplication and loss events of Physcomitrella patens, Sphagnum fallax, and Arabidopsis thaliana relative to grape, respectively. D (in red) represents duplication events and “LOST” (in gray) represents loss events. (PNG 142 kb) [file 12864_2019_5944_MOESM6_ESM.png]
